# A Hybrid Approach Combining Dual-Energy and Inpainting Methods for Metal Artifact Reduction in Dentomaxillofacial CBCT: A Proof-of-Concept Phantom Study

**DOI:** 10.1007/s10439-025-03811-1

**Published:** 2025-07-20

**Authors:** Dinidu Jayakody, Harshit Agrawal, Ella Räinä, Annina Sipola, Ritva Näpänkangas, Sampo Ylisiurua, Miika T. Nieminen, Mikael Brix

**Affiliations:** 1 Research Unit of Health Sciences and Technology, University of Oulu, Aapistie 5A, 90220 Oulu, Finland; 2 Department of Diagnostic Radiology, Oulu University Hospital, Kajaanintie 50, 90220 Oulu, Finland; 3 Research Unit of Population Health, University of Oulu, Aapistie 5A, 90220 Oulu, Finland; 4 Medical Research Center, Oulu University Hospital and University of Oulu, Oulu, Finland; 5 Research and Technology, Planmeca Oy., Asentajankatu 6, 00880 Helsinki, Finland

**Keywords:** Cone-beam computed tomography, Dual energy, Metal artifacts, Material decomposition, Virtual monochromatic imaging

## Abstract

**Purpose:**

Image inaccuracies and distortions are amplified in cone-beam computed tomography (CBCT), with beam hardening and metal artifacts being particularly pronounced, thereby complicating diagnostic interpretation. An approach, combining dual-energy CBCT based projection-domain material decomposition with virtual monochromatic imaging (VMI) technique, was leveraged to mitigate beam hardening artifacts originating from dental restorative and prosthetic materials on a diagnostic CBCT scanner in a phantom setting.

**Methods:**

Severe artifact-causing dental restorative and prosthetic materials were identified from the literature and six of them were selected for the study. Six different phantoms were developed using selected materials, and 3D-printed cylindrical molds filled with gelatine. Three different tube voltages, such as 80 kilovoltage (kV), 100 kV, 120 kV were selected for scanning and the phantoms were scanned using a commercial CBCT scanner (Viso G7, Planmeca Oy., Helsinki, Finland). A custom-developed material decomposition algorithm, based on polychromatic projection domain modeling, was employed to separate the dual-energy data into water and iron basis materials. VMIs were then synthesized at 200 keV using the decomposed data. For comparison, the 100 kV acquisition (routine protocol) with and without the vendor’s inpainting-based MAR algorithm was used to assess VMI techniques’ performance for artifact reduction.

**Results:**

Both subjectively and quantitatively, the VMI technique offered better image quality than the routine 100 kV protocol. Further, combining the VMI technique with an inpainting-based MAR algorithm offered superior artifact reduction (*p* < 0.01) for all tested materials compared to using the routine protocol and the MAR algorithm.

**Conclusions:**

The proposed VMI + MAR technique offered superior artifact reduction compared to a commercial MAR algorithm.

## Introduction

Cone-beam computed tomography (CBCT) is an imaging modality that employs a cone-shaped x-ray beam, in contrast to the fan-beam utilized in helical computed tomography (CT). CBCT specializes in generating high-resolution images, specifically designed for relatively small, designated regions such as dentomaxillofacial region. A cone-shaped x-ray beam along with a large 2D detector allows data acquisition within a single rotation around the patient yielding a simplified hardware design compared to CT, which makes CBCT a low-cost modality with three-dimensional imaging properties. CBCT has become a readily available choice for outpatient facilities due to the aforementioned benefits and the compact design of the modality.

CBCT is known for its increased scattering and reduced Hounsfield unit (HU) value accuracy [1, 2]. Additionally, imaging the dentomaxillofacial region with CBCT is often complicated by image artifacts, which produce streaks, shadows or distortions, resulting in deviations from the true underlying image. These artifacts can obscure pathological or anatomical details crucial for diagnosis and treatment planning. In dentomaxillofacial CBCT, metal artifacts (MA) result often from dental fillings, crowns, or orthodontic appliances [3].

Metal artifacts have traditionally been reduced using inpainting-based metal artifact reduction (MAR) algorithms, which extract the metal either in the reconstruction or in the projection domain, and use an inpainting approach to reduce the severity of the artifact [4]. More recently, dual-energy CT has been demonstrated as an effective method for mitigating metal artifacts through the use of a material decomposition (MD) algorithm and virtual monochromatic imaging (VMI) technique [5–8]. Furthermore, these techniques have been combined with traditional inpainting MAR for an even more effective artifact reduction [9, 10]. Even though VMI has been diagnostically adopted for helical CT, and it has been successfully combined with MAR algorithms, only a handful of studies exist on diagnostic CBCT scanners [11, 12].

The aim of this study was to first develop a dual-energy imaging technique and the necessary algorithms for CBCT context and to subsequently evaluate the effectiveness of the VMI technique in metal artifact reduction (MAR) in a simplified phantom setting. Six different phantoms were developed using commonly used dental materials that were extracted from patients during routine dentistry procedures and positioned within a 3D-printed cylindrical mold filled with gelatine. These phantoms were scanned using a clinical head and neck CBCT device and the image quality was compared between routine protocol, a dose-matched VMI technique, vendor’s MAR, and a combined VMI with vendor’s inpainting-based MAR algorithm hereafter referred to as VMI + MAR.

## Materials and Methods

### Phantoms

**Fig. 1:**
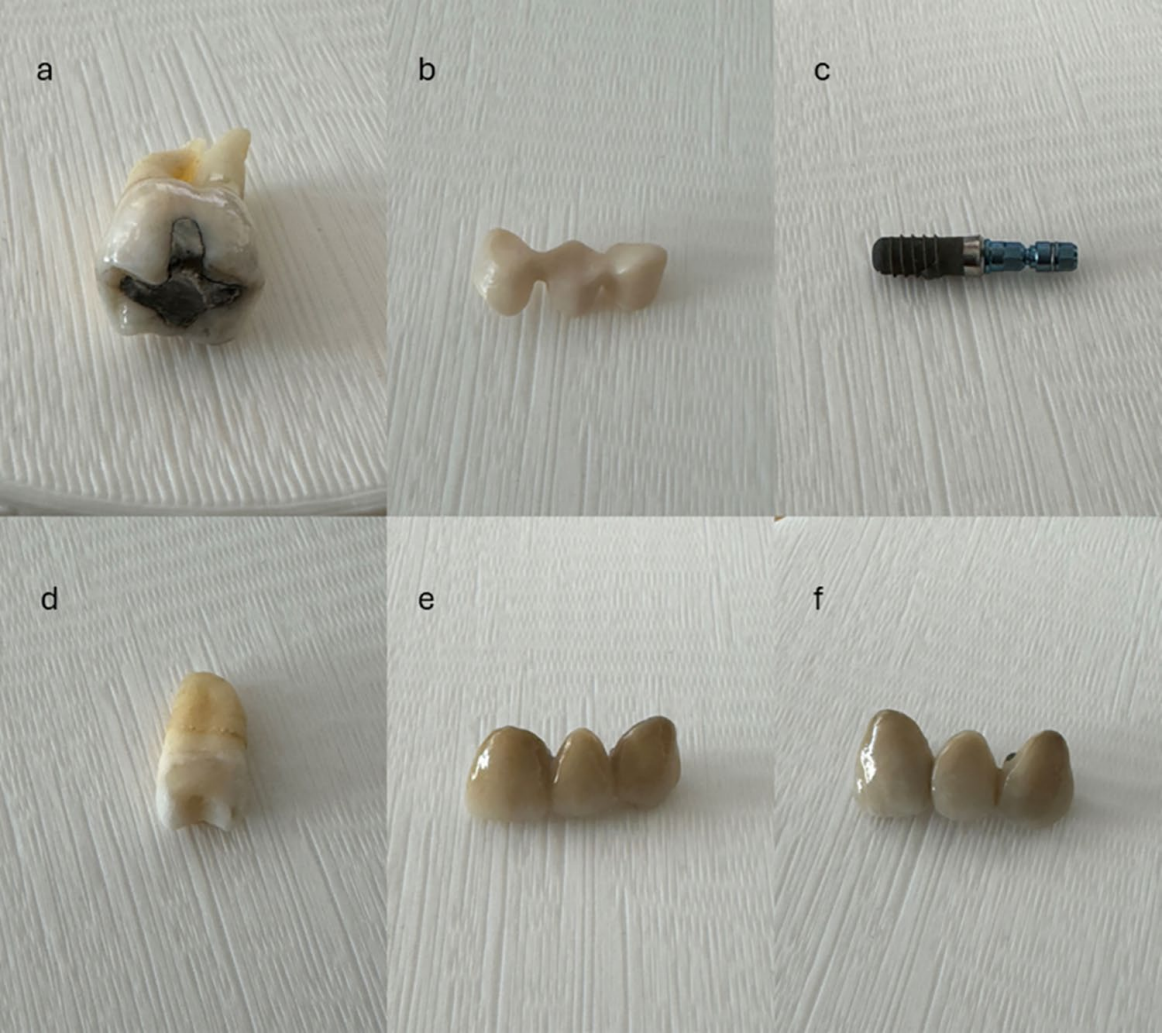
Dental materials used for the study; **a** Tooth with amalgam filling, **b** Zirconia fixed dental prosthesis (FDP), **c** Titanium dental implant, **d** Tooth with composite resin filling, **e** Cobalt-chrome alloy FDP, **f** High-gold alloy FDP

Six custom-made phantoms were developed for this study including six dental restorative and prosthetic materials identified from the literature as commonly used in the dentomaxillofacial region (Table 1) [13–17]. These materials were teeth with dental fillings, fixed dental prostheses (FDP), and dental implant which were extracted from patients during routine dental procedures (Fig. 1). The materials were received from the Department of Oral and Maxillofacial Surgery, Oulu University Hospital, Oulu, Finland. A hollow polylactic acid (PLA) cylinder (diameter 10 cm, height 10 cm) was 3D printed, and the test materials were placed in the cylinder with gelatine to mimic the real-world scenario.

**Table 1:** Dental restorative and prosthetic materials used in the study

Material	Description of the implant/prosthesis
Amalgam	Complete tooth with amalgam filling
Composite Resin	Complete tooth with composite resin filling
Zirconia	Fixed dental prosthesis
Cobalt-chrome alloy	Fixed dental prosthesis
Titanium	Titanium dental implant
High-gold alloy	Fixed dental prosthesis

This simple phantom model was utilized to avoid the interior tomography artifact [18] which would require further truncation correction. Further, the use of gelatine phantom allows for more reliable quantitative characterization as potential anatomies of the phantom do not affect the results, limiting the observed effects to be caused by the beam hardening and metal artifacts.

### Measurements

A clinical head and neck CBCT scanner (Viso G7, Planmeca Group, Helsinki, Finland) was utilized in this study. The scan settings (Table 2) were selected to be clinically relevant, and care was taken to match the dose area product (DAP) of dual-energy with the DAP of the routine scan. During preliminary testing, it was identified 9 mAs for both 80 kV and 120 kV to achieve DAP of routine (100 kV, 18 mAs)

1 $${{DAP}_{100 kV, 18 mAs}\approx DAP}_{80 kV, 9 mAs}+{DAP}_{120 kV, 9 mAs} ,$$

as shown in Equation (1).

**Table 2:** Imaging parameters of the clinical scanner

Parameter	Viso G7
Tube voltage	80, 100, and 120 kV
Filtration	Al 2.5 mm, Cu 0.2 mm
Current-time	9, 18, and 9 mAs
DAP	155, 541 and 417 mGy cm^2^
Voxel size	0.450 mm × 0.450 mm × 0.450 mm
Reconstruction size	312 × 312 × 223
Clinical protocol	Jaw
Field of view (FOV)	14 cm × 10 cm
Angular coverage	210^o^

### Material Decomposition and Virtual Monochromatic Imaging

The measured projection data from the clinical head and neck scanner were extracted and material decomposition was performed for each measured projection [19, 20]. A polychromatic material decomposition approach was used, which models the polychromatic X-ray tube spectra and the energy-dependent attenuation effects [21]. Projection-based MD models the polychromatic attenuation for a sinogram pixel *i* in different energy (kV) measurements *n* [22]:

2 $$I_{{\left( {i, n} \right)}} = \int_{0}^{{E_{{{\text{max}}}} }} {I_{{0\left( {i,n} \right)}} \left( E \right)Ee^{{ - \mathop \sum \limits_{m = 1}^{M} \mu_{m}^{{{\text{mass}}}} \left( E \right)A_{m, i} { }}} dE}$$

where ${I}_{0(i,n)}\left(E\right)$ is the number of incident X-rays with energy *E* for the X-ray spectrum *n*, and ${A}_{m,i}$ is the projected mass density along path *l*_*i*_

3 $${A}_{m,i}={\int }_{{l}_{i}}{\rho }_{m,j}ds,$$

where ${\rho }_{m,j}$ is the density of *m*-th basis material in the reconstruction voxel *j.* The MD algorithm attempts to solve the projected mass densities of selected basis materials by minimizing the l2-norm between the measurement data and the attenuation model (2). A Gauss-Newton method with 200 iterations developed with MATLAB R2022b (MathWorks, Natick, MA) was used to solve this non-linear inverse problem [21]. For this study, water and iron were selected as the basis materials, as water is the most prevalent tissue type, and due to its strong attenuation, the attenuation properties of most biological and foreign materials can be represented as a linear combination of those of water and iron [23].

The material decomposed basis projections (${A}_{\text{iron}}$_,_ ${A}_{\text{water}}$) were used to obtain the VMI projections (*p*_*E*_) as

4 $${p}_{E}={\mu }_{\text{iron}}\left(E\right){A}_{\text{iron}}+ {\mu }_{\text{water}}\left(E\right){A}_{\text{water}},$$

where ${\mu }_{\text{iron}}$, ${\mu }_{\text{water}}$ are the mass attenuation coefficients of the decomposed basis materials at selected energy (*E*). A monochromatic energy of 200 keV was selected for the study as higher VMI energies should reduce the artifacts [24].

### Reconstruction and Metal Artifact Reduction Algorithms

The 100 kV acquisition data and the virtual monochromatic projections were reconstructed using the vendor’s default Feldkamp-Davis-Kress (FDK)-based reconstruction algorithm. Additionally, both of the aforementioned projection datasets were separately deployed on the vendor’s inpainting-based MAR pipeline.

In the inpainting-based MAR pipeline, first, an initial 3D reconstruction is obtained from the 2D projections using FDK. Then, the metals are segmented in 3D using a threshold-based segmentation algorithm. The segmented metals are forward projected to obtain the metal trace in the projected domain. The pixels corresponding to the metal trace are discarded from the 2D projections and are inpainted. The inpainted projections are reconstructed again using FDK. Finally, the 3D metals obtained from the first step are put back in the inpainted reconstruction [25].

### Image Quality Assessment

**Fig. 2:**
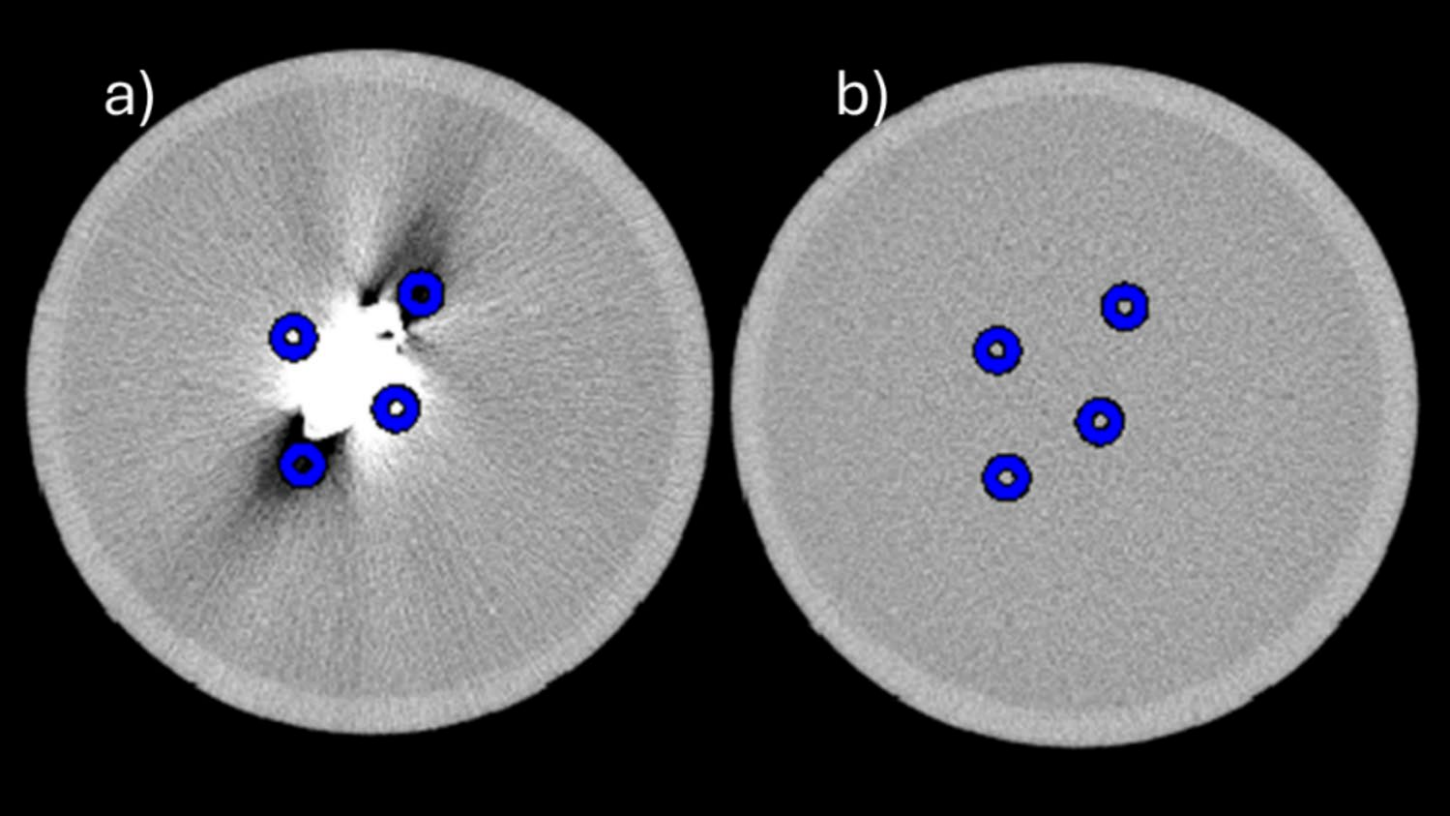
Selection of the region of interest for the quantitative analysis. Four ROIs were selected for the analysis from each selected slice. ROIs were selected as they cover the worst artifact-affected regions from the slice. **a** artifact-affected slice, **b** uniformity slice without the influence of artifact

The artifacts were addressed subjectively and quantitatively. Regarding the quantitative assessment, four regions of interest (ROIs) were selected from each artifact affected slice and ROIs were positioned to represent the most severely artifact-affected regions (Fig. 2a). Uniformity slice for the noise parameter was selected from a uniform region from the same phantom that contains only gelatine (Fig. 2b).

5 $$\text{Artifact score}= \left|\overline{{ROI }_{\text{Artifact}}}- \overline{{ROI }_{\text{Uniformity}}}\right|$$

Artifact score was calculated using equation 5 for routine reconstruction, VMI 200 keV, vendor’s MAR, VMI + MAR, and compared to identify the performance of each method. $\overline{{ROI }_{\text{Artifact}}}$, $\overline{{ROI }_{\text{Uniformity}}}$ indicate the mean values of ${ROI}_{\text{Artifact}}$ and ${ROI}_{\text{Uniformity}}$ respectively. Absolute value is used to summarize the effect, as in some regions the artifact is seen as intensity loss and in others as intensity gain. A lower artifact score indicates better artifact reduction.

### Statistical Analysis

Statistical significance between tested four methods (100 kV, VMI, Inpainting MAR, VMI + MAR) was evaluated using the paired T-test. Slice-wise artifact scores for each method from the exact same slice from all six materials were aggregated and pairwise comparisons between methods were conducted. *P*-values were calculated from each pairwise comparison, with statistical significance set at *p* < 0.01.

## Results

**Fig. 3:**
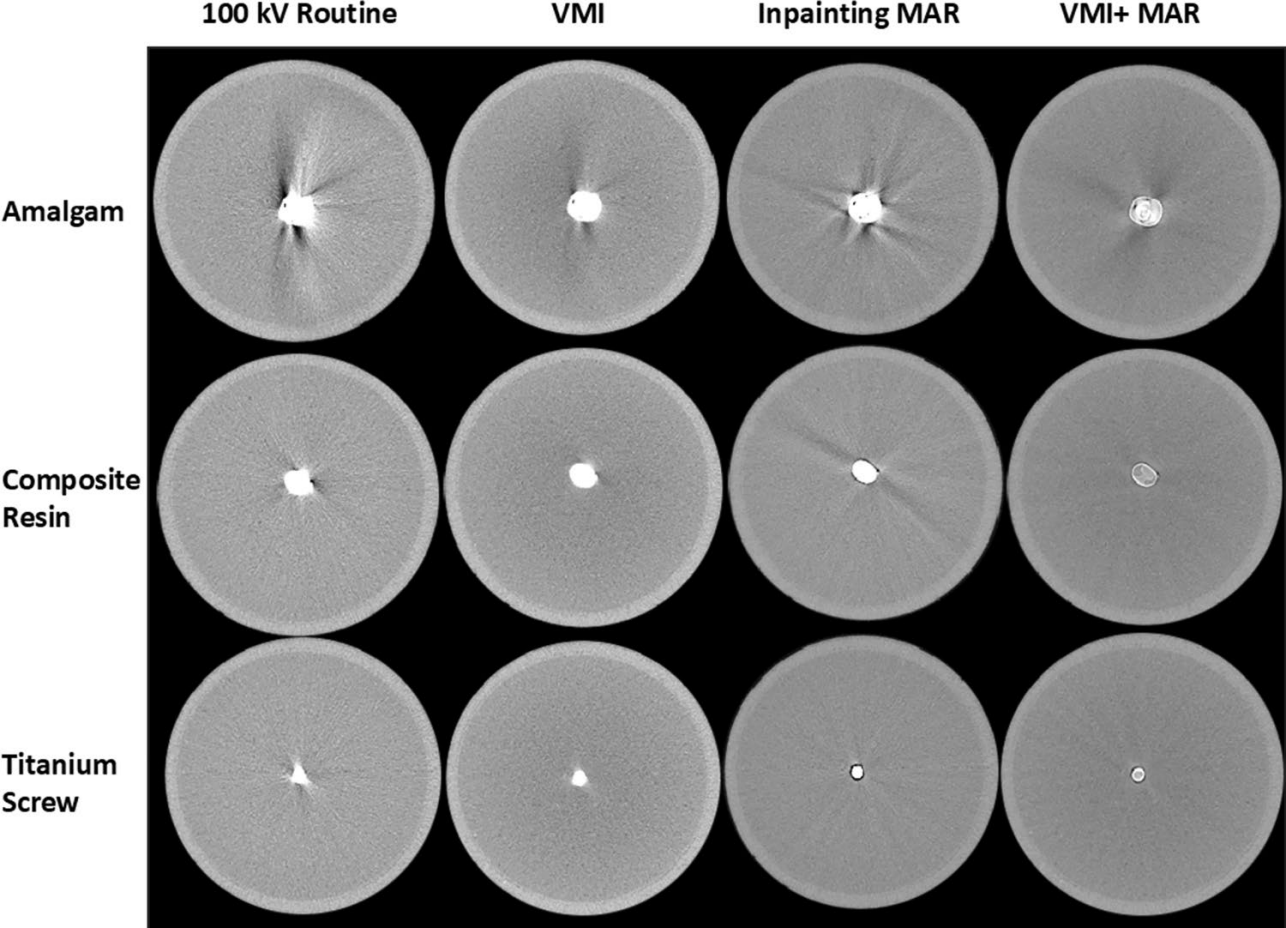
Axial view of the different reconstructions of teeth with amalgam and composite resin fillings, and titanium dental implant. As the VMI technique affects Hounsfield Unit values, the windowing was adapted for each implant separately using a mean-centered windowing technique, which adjusts the intensity range based on a defined ROI

**Fig. 4:**
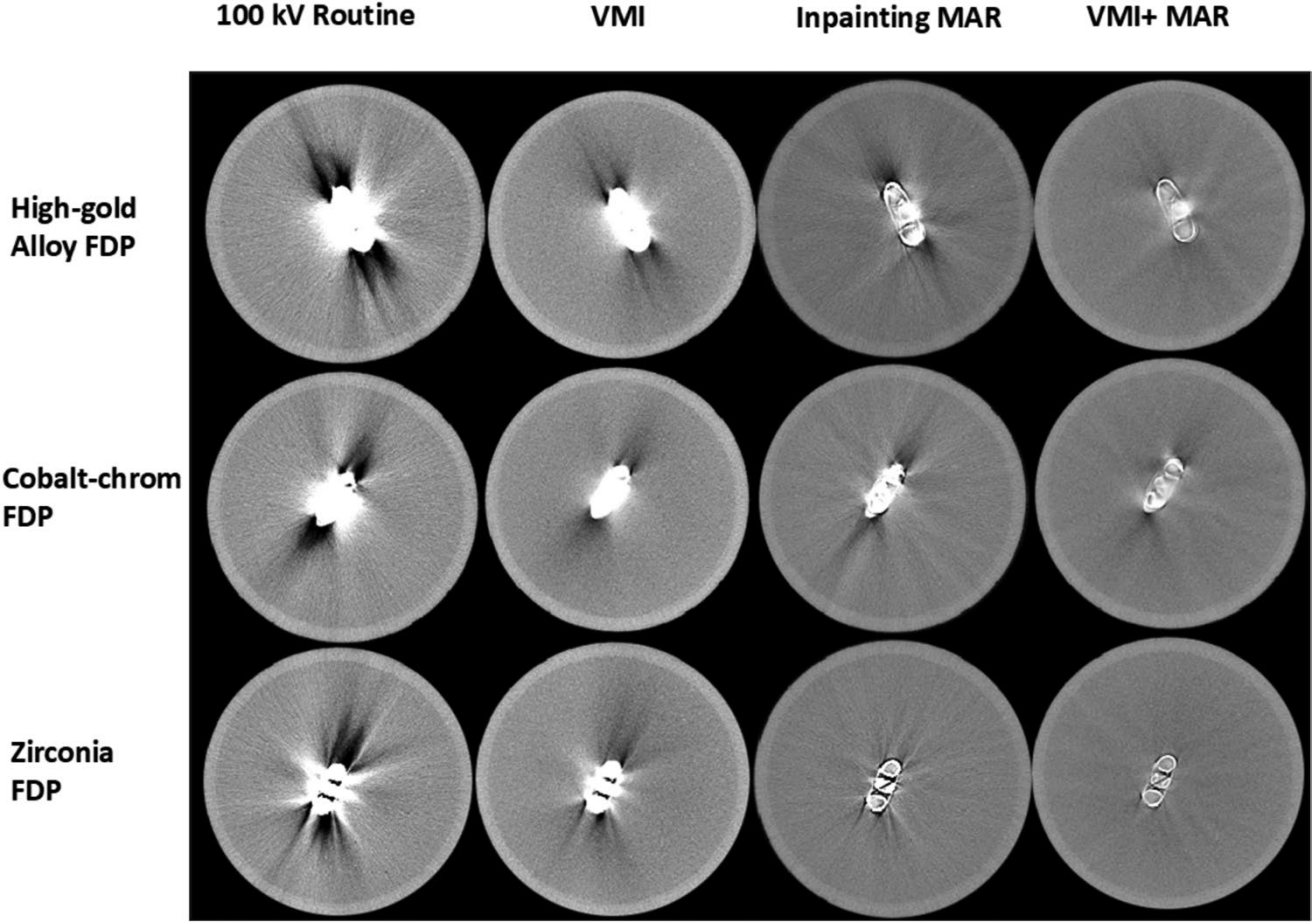
Axial images of fixed dental prostheses (FDPs)

**Fig. 5:**
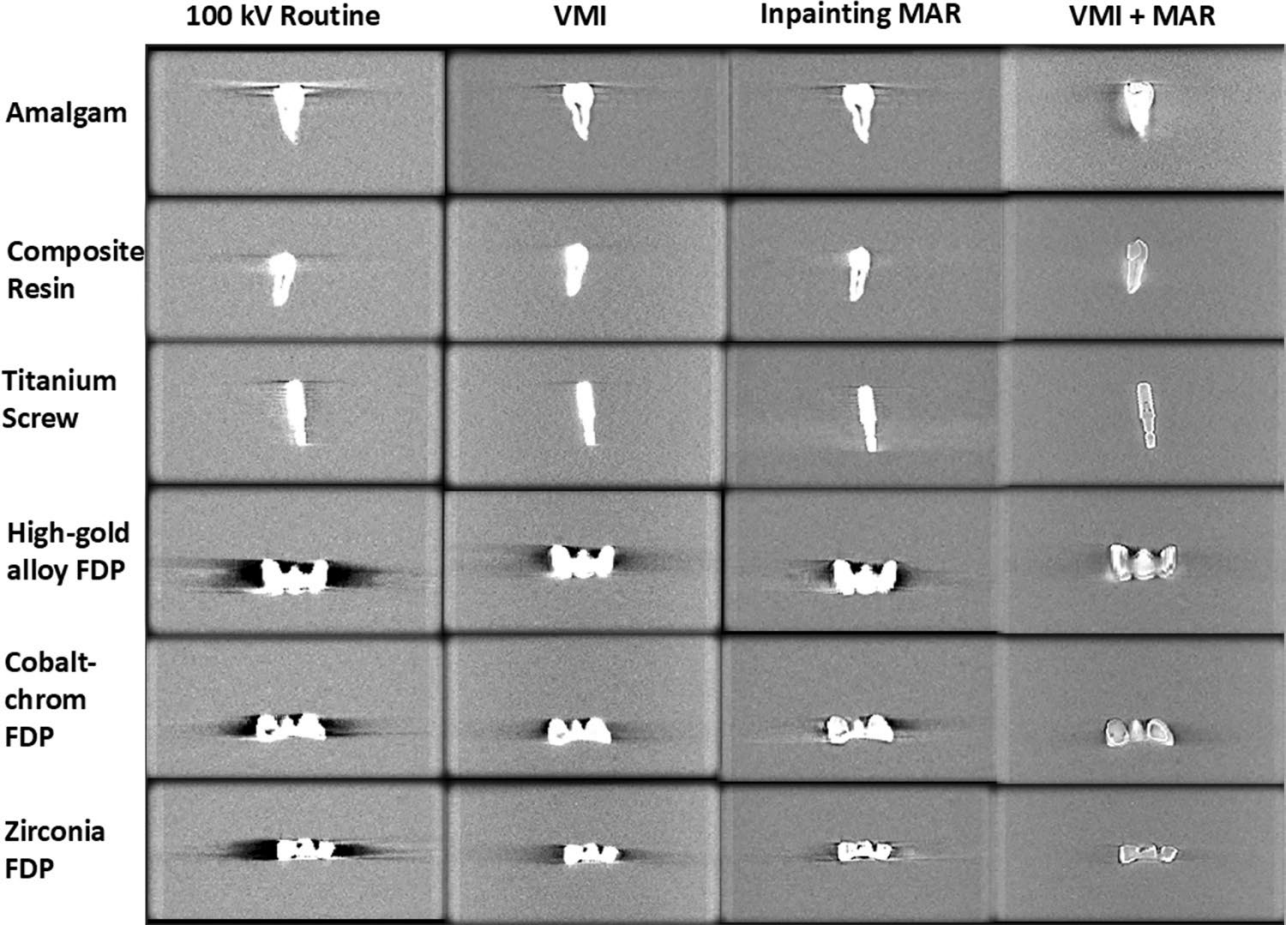
Visualization of the dental restorative and prosthetic materials along the metal artifacts progression direction. The improved visualization of dental material structure is not only due to windowing level as, e.g., the titanium implant screw structure could not be characterized with any windowing setting using other methods than VMI + MAR

VMI and the inpainting MAR significantly reduce the metal artifacts compared to the routine 100 kV reconstruction (Fig. 3). VMI may leave some trace of the original artifact (seen in the 100 kV image) in the VMI reconstruction, but it does not generate artifacts in other directions. Inpainting MAR, on the other hand, is often powerful in the direction of the most severe artifact, but it can induce stripe-like artifacts in new directions. Combining VMI with MAR resulted in superior artifact reduction. The effect of the different reconstructions was clearly visible in the fixed dental prostheses which were observed to be the worst artifact generation (Fig. 4). Visualization along the direction of the most severe artifact demonstrates the VMI + MAR method not only mitigates the metal artifacts, but also preserves the image quality, enabling the visualization of the internal structures of the restorative and prosthetic materials (Fig. 5) and preserving the surrounding region.

**Fig. 6:**
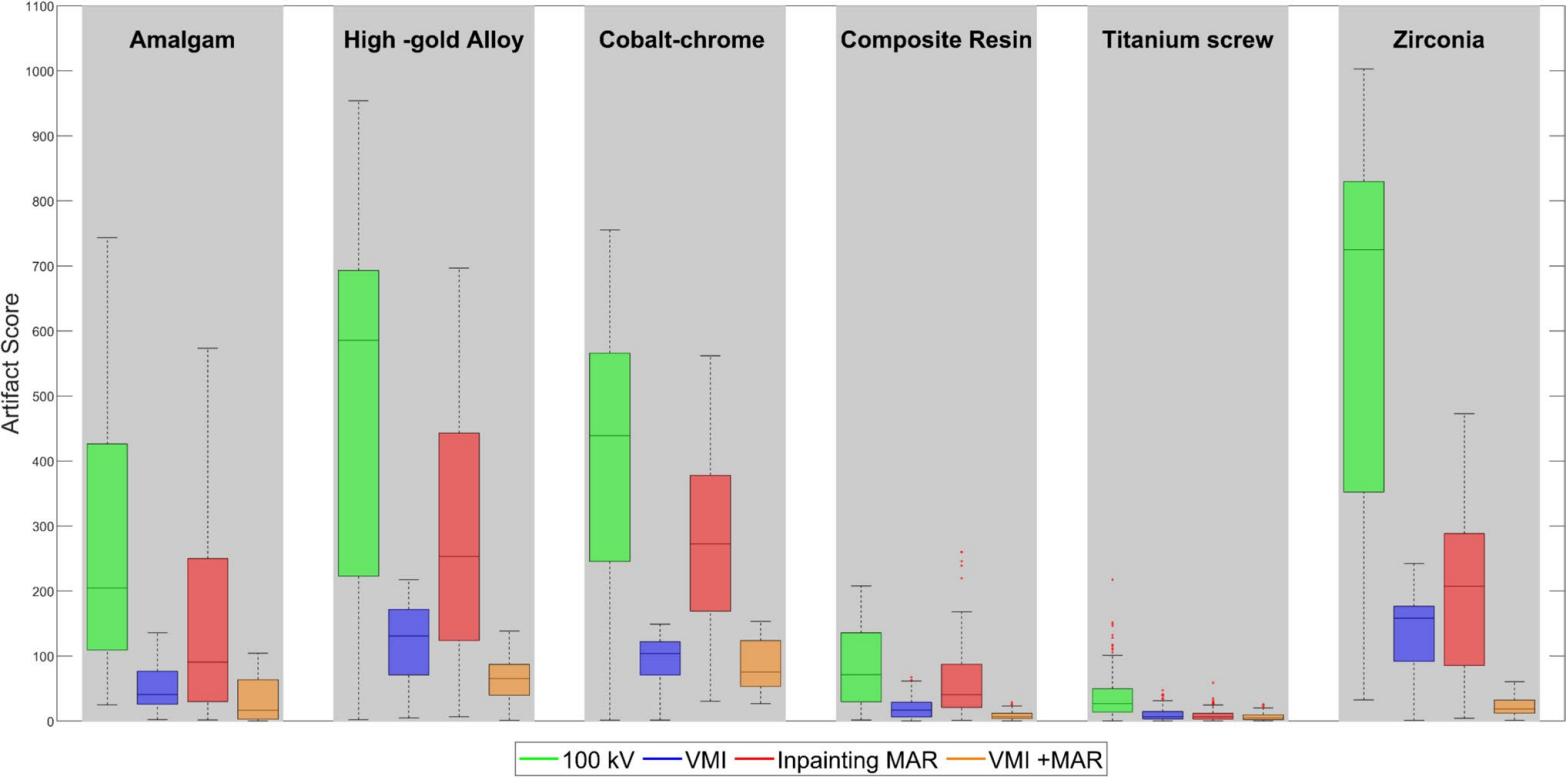
Quantitative comparison of slice-wise artifact scores of all reconstruction techniques. Lower artifact score values indicate better metal artifact reduction

The quantitative analysis represents the slice-wise artifact scores obtained using (5). The low artifact scores affirm the effectiveness of using VMI and the even superior performance with VMI + MAR. Both methods outperform the commercial MAR for all cases (Fig. 6) and there is a statistically significant difference between each method (Table 3), indicating a significant reduction in artifacts.

**Table 3:** *P*-values obtained from paired comparison between four tested methods for aggregated data from all six materials

	100 kV	VMI	Inpainting MAR	VMI + MAR
100 kV	1	< 0.01 (+)	< 0.01(+)	< 0.01 (+)
VMI	< 0.01 (−)	1	< 0.01 (−)	< 0.01 (+)
Inpainting MAR	< 0.01 (−)	< 0.01 (+)	1	< 0.01 (+)
VMI + MAR	< 0.01 (−)	< 0.01 (−)	< 0.01 (−)	1

Directionality is denoted by (+) or (−), where (+) indicates better performance and (−) indicates inferior performance in terms of artifact reduction. For example, when we consider 100 kV against VMI (row 2, column 3 in the table) *p* < 0.01 (+), which means there is a significant difference between the artifact scores of 100 kV acquisition and VMI. Since the directionality is positive (+), the artifact reduction in VMI is better than 100 kV. Other way around, if we evaluate VMI against 100 kV (row 3, column 2 of the table), *p* < 0.01 (−), which means there is a significant difference between two sets and VMI is better, and 100 kV is inferior in artifact reduction

## Discussion

This study investigated the effectiveness of the dual-energy CBCT for metal artifact reduction in the dentomaxillofacial region. Polychromatic material decomposition and virtual monochromatic imaging techniques were deployed with commercial FDK and MAR algorithms to study whether the method yields improved suppression of metal artifacts.

In our preliminary tests, we observed that monochromatic material decomposition approaches [26] can separate soft tissues and bone accurately, but fail to decompose metals accurately. When performing the MD, a monochromatic approximation of the beam considers the effective energy of the polychromatic beam for the calculations [26]. Due to the strong beam hardening caused by dental implants, monochromatic MD did not provide the optimal performance and thus polychromatic modeling of the spectra was required. This was an interesting observation as we have internally observed that the beam hardening from bones can already be reduced by combining monochromatic MD with the VMI algorithm. Apparently, however, with strongly attenuating dental materials polychromatic model is needed.

The current study employed a simplified two-material decomposition model used water and iron as the basis materials, which is a common approach in dual-energy CT frameworks due to its computational efficiency and feasibility in clinical implementations. However, this model may not fully characterize the complex compositions of dental prosthetic and restorative materials, which often include wide array of high atomic numbers such as amalgam, zirconia, and high-gold alloy. These varying compositional characteristics could significantly alter the X-ray attenuation properties and, consequently, the accuracy of the material characterization and artifact reduction [27].

Extending to a multi-material decomposition framework such as a tri-material approach could improve the accuracy of the material decomposition in a heterogeneous clinical context [28]. These advanced models may enable better accommodation of overlapping spectral properties and provide more precise quantification by leveraging additional material-specific information or prior knowledge of the scanned object [29]. While integration of such approaches presents an interesting opportunity, it was considered out of scope of this study, as dual-energy techniques are typically combined with dual-material decomposition.

Six different dental restorative and prosthetic materials were used in this study. It was observed that resin composite demonstrated the least visible artifacts likely due to the low effective atomic number of the material compared to e.g., zirconia. The titanium implant screw did not cause severe artifacts owing to its smaller size. Tooth with amalgam filling generated severe artifacts and all the fixed dental prostheses (cobalt-chrome alloy, high-gold alloy, and zirconia) exhibited substantial artifacts. Zirconia was identified as the most severe source of artifacts (Figs. 3, 4, 5). The developed VMI and VMI + MAR methods were superior as compared to 100 kV reconstruction and commercial MAR for all tested dental filling materials and prostheses.

While MD in the projection domain effectively reduces beam hardening, it is crucial to consider the radiation dose experienced by the patient. DAP values were 155, 541, and 417 mGy cm^2^ for 80, 100 and 120 kV respectively. The DAP values were matched with 100 kV routine acquisition to prevent additional doses to make the approach clinically feasible. However, as the exposure values cannot be freely chosen, and can instead be changed within a pre-specified set of values, the DAP values could not be exactly matched and the dose of the DE-CBCT acquisition was 5.7% higher.

VMI + MAR limited the artifact into a smaller region, which may indicate that this could help in detecting lesions closer to the metals. However, further studies are needed to evaluate this prospect. Furthermore, interior tomography issue, in which the radiation beam covers only a part of the whole object in the axial plane [30], is often encountered in dentomaxillofacial CBCT. The VMI technique has proven to be effective in mitigating the beam hardening arising from bones located outside the FOV in the cardiac imaging context [31]. Due to the dense bone structures present in the dentomaxillofacial region, these beam-hardening effects originating from strongly attenuating regions outside the FOV are prevalent in dentomaxillofacial CBCT. Consequently, the presented approach could also reduce these artifacts in the dentomaxillofacial region.

In this study, we selected the absolute difference in mean intensity between artifact-affected and uniformity ROIs as our primary artifact quantification metric. This method is often used in the literature to evaluate the metal-induced CT number deviations [32, 33]. Metrics such as contrast-to-noise ratio (CNR) have been shown to lack robustness in metal artifact quantification [34]. Spatial resolution was not used as a quantitative metric in this study since the reconstruction kernel used was the same for all MAR techniques utilized. Hence, the overall resolution of the reconstructed images should not deteriorate between the routine reconstruction and the VMI techniques, and no resolution target was positioned near the metal. However, smoothening near the metal objects has been reported for inpainting-based MAR methods [35]. To quantitatively address those effects, future studies could incorporate resolution assessment using task-transfer function [36] and dedicated anthropomorphic phantoms with inserted metals, for instance.

One limitation of this study is that sequential data acquisition was incorporated. Obtaining low-energy and high-energy acquisitions separately in a clinical setting may lead to misregistration due to patient motion. Resulting temporal misalignments could adversely affect the accuracy of the material decomposition, eventually posing potential major effects on quality of the VMI, especially in regions like orofacial structures [37]. Misregistration may lead to inconsistencies in projection data resulting in inaccuracies in spectral decomposition, artifact propagation, and compromised quantification reliability [27]. Advanced motion correction algorithms have been proposed to mitigate these issues, including multi-resolution, mutual information-based registration techniques that operate at both global and local anatomical scales [38]. Additionally, dual-energy imaging may benefit from utilizing fast kVp-switching or dual source systems, which enables acquiring both energy datasets nearly simultaneously. This could significantly reduce the susceptibility to motion-related artifacts and improve spectral accuracy within a reduced acquisition time [39]. 

Another suggestion would be to use a dual-layer detector system in CBCT, in which the first scintillation layer is sensitive for low-energy photons, while the second scintillation layer is optimized for high-energy photon detection. Detector layer arrangement geometry minimizes the misregistration associated with sequential acquisitions [40].

Photon counting detectors (PCDs) could be another highly effective method for DE cone-beam computed tomography since they allow accurate separation of X-ray photons based on their energy, allowing for dual-energy or multi-energy CBCT. This technology would capture separate energy spectra from a single exposure [41]. Additionally, PCDs would remove electronic noise, provide more optimal energy weighting increase contrast, and allow for improved detection efficiency [42–44].

As future work, the utility of PCDs for DE-CBCT will be addressed. Furthermore, anthropomorphic phantoms and out-of-FOV positioning of the artifact sources will be addressed to evaluate the performance of the method in a more diagnostically representative scenario. Our attempts to extend this study to an anthropomorphic setting did not succeed since the phantoms available for us did not allow placing insets inside the dentomaxillofacial area. Alternatively, we tried taping the materials onto the surface of the phantom. For this scenario, the inpainting MAR failed in artifact reduction since it did not represent a natural imaging scenario, in which the metal is inside the patient. Hence, we did not include anthropomorphic phantoms in this study. However, owing to the promising results, we are planning to continue toward procuring a phantom that can be drilled as future work. As the dual-acquisition is not routine with patients, we cannot perform retrospective data collections, but would need to conduct a proper clinical trial, which was not considered appropriate at this technology readiness level.

Even though this study compares the proposed dual-energy VMI approach with vendor’s inpainting based MAR algorithm, it would be important to assess its performance relative to other advanced techniques. Iterative MAR (iMAR) methods, for example have demonstrated superior artifact reduction and HU accuracy over traditional inpainting-based MAR techniques in both phantom and clinical settings, particularly around metallic implants like hip prosthesis and dental fillings [45]. These methods incorporate projection-domain corrections and iterative reconstructions models to reduce photon starvation and beam hardening effects more effectively.

Artificial intelligence-based recent MAR techniques have also offered promising results through data-driven learning from paired artifact-corrupted and corrected images. Particularly, networks like InDuDoNet use dual-domain architecture to incorporate both image and sinogram information, achieving substantial improvements in artifact reduction [46]. However, these methods require large, annotated datasets and complex training protocols.

Dual-energy VMI remains a practical and widely accessible method. It is particularly effective for beam hardening correction and is increasingly used in combination with iterative methods to achieve optimal image quality [47]. In this study, our approach provides a feasible balance between clinical applicability and artifact reduction, enabling the technique to be readily incorporated into the commercially available inpainting-based MAR of the vendor. Future studies should explore the aforementioned hybrid models that integrates VMI with iterative or AI-based MAR techniques for more efficient artifact mitigation.

Considering the promising results, integrating the DE-CBCT method in commercial CBCT would be beneficial in terms of mitigating the MAs and potentially in enhancing the material characterization as well [48].

The findings of this study indicate that dual-energy-based CBCT combined with material decomposition and virtual monochromatic imaging technique effectively reduces the metal artifacts in cone-beam computed tomography. Combining the VMI technique with an inpainting MAR produced a particularly effective artifact reduction technique.

## Data Availability

Not applicable.
